# Physicochemical Properties of the Soluble Dietary Fiber from *Laminaria japonica* and Its Role in the Regulation of Type 2 Diabetes Mice

**DOI:** 10.3390/nu14020329

**Published:** 2022-01-13

**Authors:** Xixi Wang, Liping Zhang, Ling Qin, Yanfeng Wang, Fushan Chen, Changfeng Qu, Jinlai Miao

**Affiliations:** 1 College of Chemistry and Molecular Engineering, Qingdao University of Science and Technology, Qingdao 266042, China; WXi1173596132@163.com (X.W.); chenfushan@qust.edu.cn (F.C.); 2 Key Laboratory of Marine Eco-Environmental Science and Technology, First Institute of Oceanography, Ministry of Natural Resource, Qingdao 266061, China; 15689487176@163.com (L.Z.); qinlingouc@163.com (L.Q.); yanfengw123@163.com (Y.W.); cfqu@fio.org.cn (C.Q.); 3 Department of Special Medicine, School of Basic Medicine, Qingdao University, Qingdao 266000, China; 4 Laboratory for Marine Drugs and Bioproducts, Qingdao Pilot National Laboratory for Marine Science and Technology, Qingdao 266237, China

**Keywords:** dietary fiber, *Laminaria japonica* byproducts, diabetes, intestinal flora, intestinal metabolites

## Abstract

*Laminaria japonica* is a large marine brown alga that is annually highly productive. However, due to its underutilization, its potential value is substantially wasted. For example, a lot of *Laminaria japonica* cellulose remains unused during production of algin. The soluble dietary fiber (SDF) was prepared from the byproducts of *Laminaria japonica*, and its physicochemical properties were explored. SDF exhibits good water-holding, oil-holding, water-absorbing swelling, glucose and cholesterol absorption capacity, and inhibitory activity of α-amylase and α-glucosidase. In addition, the beneficial effects of SDF in diabetic mice include reduced body weight, lower blood glucose, and relieved insulin resistance. Finally, the intestinal flora and metabolomic products were analyzed from feces using 16S amplicon and LC-MS/MS, respectively. SDF not only significantly changed the composition and structure of intestinal flora and intestinal metabolites, but also significantly increased the abundance of beneficial bacteria *Akkermansia*, *Odoribacter* and *Bacteroides*, decreased the abundance of harmful bacteria *Staphylococcus*, and increased the content of bioactive substances in intestinal tract, such as harmine, magnolol, arachidonic acid, prostaglandin E2, urimorelin and azelaic acid. Taken together, these findings suggest that dietary intake of SDF alleviates type 2 diabetes mellitus disease, and provides an important theoretical basis for SDF to be used as a functional food.

## 1. Introduction

Type 2 Diabetes Mellitus (T 2DM) is a complex metabolic disease characterized by hyperglycemia that is prone to a series of complications. According to the data released by the International Diabetes Federation (IDF) in 2019, approximately 463 million adults aged 20–79 years suffer from diabetes, and this number is expected to reach 578.4 million by 2030 and reach 700.2 million by 2045 [1].

Therefore, exploring the treatment of diabetes has always been the focus of all countries in the world. There are as many as 3 million genes carried by microbes in the human gut, of which more than 99% belong to bacteria [2]. Firmicutes and bacteroides occupy the majority of the intestinal tract, accounting for 60–80% and 15–20%, respectively, and their proportion is closely related to metabolic diseases in the body [3]. The metabolic capacity of intestinal flora greatly exceeds human cells, and the bioactive metabolites produced directly or indirectly affect the physiological function of the host [4]. For example, *Blautia*, *Roseburia* and *Turicibacter* produce butyric acid [5,6], and butyric acid improves intestinal permeability, increases insulin sensitivity, and relieves inflammation and glucose intolerance [7]. Consequently, discovering and deciphering the interrelationships between gut flora, metabolites, and hosts may be a new approach for the prevention and treatment of metabolic diseases, such as diabetes.

Although the hypoglycemic effect of commercially available hypoglycemic drugs is remarkable, paying attention to diet can not only improve physical fitness, but also assist drugs to treat diabetes [8]. For example, taking a certain amount of dietary fiber can help to lower blood lipids and maintain blood glucose. The dietary fiber of terrestrial plants, such as cereals, fruits and vegetables, were studied more than algae. *Laminaria japonica* has high nutritional value, is rich in algin, dietary fiber and minerals, and is a good source of dietary fiber production. China is a major *Laminaria japonica* producer, with an annual output of more than 300,000 tons [9,10], but approximately 50% of *Laminaria japonica* is wasted due to insufficient utilization. *Laminaria japonica* byproducts contain many nutritional ingredients, and once they are discharged into the ocean, they cause great pressure on the marine environment and result in the formation of red tide and other hazards [11]. To make full use of *Laminaria japonica* resources, many beneficial natural products are extracted from *Laminaria japonica*, such as *Laminaria japonica* polysaccharides, which reduce blood glucose and prevent hypertension, cardiovascular and cerebrovascular diseases and renal failure [12]. Dietary fiber is a polysaccharide that cannot be digested or absorbed by the human small intestine, but it may be completely or partially fermented in the large intestine to regulate the abundance and metabolites of intestinal microorganisms.

*Laminaria japonica* is an important large-scale economic alga in algae cultivation that is a marine food vegetable rich in algin, mannitol, iodine and other nutrients, and is important raw material for medical, health care and agricultural fertilizer industries. However, a large amount of cellulose in *Laminaria japonica* byproduct was neglected after the align production. To improve the utilization rate of *Laminaria japonica* and explore the economic value of *Laminaria japonica* in industry and food processing, SDF was first extracted from the waste *Laminaria japonica* byproducts after algin extraction, and its physicochemical properties and glucose-lowering effect were studied in db/db mice. In addition, the classical 16S rRNA gene sequencing analysis method was used to detect the intestinal flora of mice, and to explore the regulatory effect of SDF on the composition and structure of intestinal microflora in diabetic mice. Other studies on intestinal metabolites of SDF mainly focused on short-chain fatty acids, while this study screened out six beneficial metabolites through non-metabolic studies (HPLC-MS/MS), laying a foundation for further exploring the potential mechanism of intestinal action of SDF and utilization of *Laminaria japonica* resources.

## 2. Materials and Methods

### 2.1. Reagents and Materials

The *Laminaria japonica* byproducts used to produce soluble dietary fiber were provided by Yantai Intercontinental Marine Biotechnology Co., Ltd. (Yantai, Shandong, China), which was the algae debris after the production of algin. The monosaccharide and dextran standards were purchased from Sigma-Aldrich (St. Louis, MO, USA). DNS reagent, α-glucosidase α-amylase, p-nitrophenyl-α-D-glucopyranoside, OPA reagent, triglycerides cholesterol (TG), total cholesterol (TC), high-density lipoprotein cholesterol (HDL-c), low-density lipoprotein cholesterol (LDL-c) and insulin (INS) detection kits were purchased from Beijing Zhong Sheng Beikong Biotechnology Co., Ltd. (Beijing, China). Experimental methanol, formic acid, and ammonium acetate for intestinal metabolomics were purchased from Thermo Fisher Scientific Co. Ltd. (Waltham, MA, USA). The basic diet was purchased from Jiangsu Xietong Pharmaceutical and Biological Engineering Co., Ltd. (Nanjing, Jiangsu, China). All chemicals and solvents were analytical-grade or HPLC-grade.

### 2.2. SDF Preparation

The byproducts after extracting algin from *Laminaria japonica* mainly contain insoluble polysaccharide, so the SDF extraction method in this study was different from the traditional SDF extraction method, and the extraction method of SDF was modified according to the previous report [13]. The mixture of *Laminaria japonica* byproducts and distilled water was boiled for 30–50 min, and the pH was adjusted by 5 M sodium hydroxide solution. The mixture was stirred at 90 °C for several hours, centrifuged to retain the supernatant, and pH = 7 was adjusted with 50% hydrochloric acid. The adjusted supernatant was concentrated in a rotary evaporator and added to a 1500 Da dialysis bag for 10–12 h, and then placed in a freeze dryer for 36 h to obtain dry SDF.

### 2.3. Functional Properties

#### 2.3.1. Water-Holding Capacity (WHC)

According to the modified method [14], the 1 g SDF was added to 25 mL of distilled water, stirred at room temperature for 30 min, and then centrifuged at 5000 r/min for 10 min in a centrifuge. After the supernatant was removed, the water on the centrifuge tube wall was suctioned through a filter paper, the weight was recorded M_1_, after drying at 105 °C to constant weight, and then the weight was recorded M_2_. WHC was calculated by the following formula: WHC (g/g) = (M_1_ − M_2_)/M_2_.

#### 2.3.2. Oil-Holding Capacity (OHC)

According to the method and make modifications [15], we accurately weighed 1 g SDF as M_1_, and the SDF was added 10 mL of corn oil, stirred continuously for 30 min at room temperature, centrifuged in a 5000 r/min for 10 min, removed the supernatant, drained the excess oil on the centrifugal tube wall with filter paper, and then it was weighed as M_2_. Additionally, the following formula was used to calculate OHC: OHC (g/g) = (M_2_ − M_1_)/M_1_.

#### 2.3.3. Water-Swelling Capacity (WSC)

According to the methods with modifications [16], 200 mg of SDF was accurately weighed and expressed as M, placed in a 10 mL glass measuring cylinder, and 5 mL of distilled water was accurately added, and the liquid level height was recorded as V_1_. After being stirred and mixed, it was placed at room temperature for 24 h, and the liquid level height was recorded as V_2_. WSC was calculated using the following formula: WSC (mL/g) = (V_2_ − V_1_)/M.

#### 2.3.4. Glucose Absorption Capacity (GAC)

According to the literature method, the standard curve of glucose was drawn [17]. We accurately weighed 0.1 g SDF as M, while the blank control group was not given SDF, and the glucose solution (10 mL, 0.1 mmol/mL, pH = 7) was added, respectively. After mixing, it was stirred at 37 °C for 6 h and centrifuged at 3000 r/min for 20 min. After adding 2 mL DNS, it was fully mixed and boiled in boiling water for 5 min, cooled to room temperature, and then the absorbance was detected by OD_540_ in the ultraviolet spectrophotometer. The concentration of glucose solution was calculated based on the standard curve. The glucose content of the blank group was expressed as A, and the glucose content added with SDF was expressed as B. The glucose absorption capacity of SDF was calculated following the formula: GAC (mmol/g) = (A − B)/M.

#### 2.3.5. Cholesterol Adsorption Capacity (CAC)

The standard curve of cholesterol was drawn according to the method in the literature [18]. After separating ordinary egg yolks, we diluted them with 9 times the volume of deionized water and beat them thoroughly. Then, taking 15 mL stirred yolk solution, we added 50 mg SDF, adjusted pH to 2 and 7, respectively, to simulate the stomach and intestinal environment (the solution without SDF was blank control), stirred at 37 °C for 2 h, centrifuged at 4000 r/min for 20 min to retain supernatant. The 1.5 mL of the OPA reagent and 1 mL of concentrated sulfuric acid were added into 400 μL supernatant, respectively, which were mixed well and developed color at room temperature for 10 min. The absorbance was measured using OD_550_ of the ultraviolet spectrophotometer, and the cholesterol concentration was calculated according to the standard curve. The cholesterol content of blank control was expressed as A, the cholesterol content after SDF adsorption was expressed as B, and the added weight of SDF was expressed as M. The formula of SDF adsorption cholesterol was as follows: the CAC (mg/g) = (A − B)/M.

### 2.4. Monosaccharide Composition and Molecular Weight of SDF

The monosaccharide composition of SDF was performed according to the method of previous research [19]. The molecular weight of SDF was detected by Gel Permeation Chromatography (GPC) with dextran as the standard. 50 mg SDF was dissolved in a certain amount of ultrapure water, and the SDF solution was stirred for 12 h, adjusted to 25 mL, and injected through a 0.22 μm filter. The detectors were Wyatt Dawn Heleos11 and Optilab T-Rex, the chromatographic column was Shodex OHpak SB-806HQ in series with SB-804HQ, and the mobile phase was ultrapure water (0.02% sodium azide) with PH = 6. The molecular weight measurement range was 2–20 million Da, the column temperature was 25 °C, and the flow rate was 1.0 mL/min.

### 2.5. Inhibition of α-Amylase Activity by SDF

The α-amylase activities inhibitory activity was determined as previously described with minor modifications [20]. The α-amylase activity was inhibited by SDF solution or acarbose solution at different gradient concentrations (0.2, 0.4, 0.6, 0.8 and 1 mg/mL). Glucose concentration was measured by the dinitrosalicylic acid method. The inhibition rate of α-amylase activity was defined as the reduction rate of glucose production in the presence of SDF or acarbose compared with the blank group. The following equation: Inhibition rate (%) = (A_2_ − A_1_)/A_2_ × 100, where A_1_ was the glucose concentration in the presence of SDF or acarbose, and the A_2_ was the glucose concentration in the blank group.

### 2.6. Inhibition of α-Glucosidase Activity by SDF

The inhibition of α-glucosidase activity was measured using a previously reported method with slight modifications [21]. Briefly, 20 μL sample (SDF or acarbose, 0.2, 0.4, 0.6, 0.8 and 1 mg/mL) and 40 μL α-glucosidase (0.1 Unit/mL) were added to the 96-well plate, and the mixture was kept in a 37 °C incubator for 10 min. Added 40 μL of p-nitrophenyl-α-D-glucopyranoside (pNPG, 1 mmol/L), and incubated at 37 °C for 30 min. Finally, we added 100 μL Na_2_CO_3_ (0.2 mol/L) to stop the reaction, and determined the OD_405_ value. The potassium phosphate buffer (pH = 6.8) was used as the solvent for all reagents and samples. We calculated the inhibition (%) by using the following equation: inhibition (%) = (A_2_ − A_1_)/A_2_ × 100, where A_1_ was the OD_405_ value in the presence of SDF or acarbose, and the A_2_ was the OD_405_ value in the blank group.

### 2.7. Animal Treatments and Sample Collection

The db/db mice and BKS wild-type mice were fed adaptively for 7 days with free water and diet. The temperature in the animal room was stable at 23–24 °C, the relative humidity was maintained at 60–65%, and a 12 h light/dark cycle was employed. Mice were randomly divided into 3 groups (*n* = 7) after one week of adaptation, wherein the model control group (dbH_2_O) and the normal control group (Normal) were gavaged with 0.1 mL/10 g body weight of purified water, and the soluble *Laminaria japonica* dietary fiber group (dbSDF) was gavaged with 0.05 g SDF/10 g body weight. The experiment lasted for 5 weeks, and mice feces were collected and stored at −80 °C immediately in the last week. Oral glucose tolerance test was performed according to the method described in the previous experiment [22]. At the end of the experiment, all mice were anesthetized and sacrificed after overnight fasting. Serum was collected and tested for TG, TC, HDL-c, LDL-c, and INS according to the instructions. All animal experimental procedures were proved by the Institutional Animal Care and Use Committee, Ocean University of China (Certificate No. SYXK20120014).

### 2.8. Extraction of Genome DNA and Amplicon Generation

The CTAB/SDS method was used to extract the total genomic DNA of the fecal sample. The specific experimental methods are consistent with the previously published articles in our laboratory [23]. The CTAB/SDS method was used to extract the total genomic DNA of the fecal sample. According to the DNA concentration, it was diluted to 1 ng/μL with sterile water and purified with 1% agarose gel. The V_3_–V_4_ hypervariable regions of the bacteria 16S rRNA were amplified using specific primer (515F-806R) with the barcode. The PCR temperature cycles were as follows: 98 °C for 1 min, followed by 30 cycles, 98 °C for 10 s, 50 °C for 30 s, 72 °C for 30 s, and finally 72 °C for 5 min. PCR reactions were carried out with 15 μL of Phusion**^®^** High-Fidelity PCR Master Mix (New England Biolabs) containing 1 μM of each primer and about 10 ng template DNA. The TruSeq**^®^** DNA PCR-Free Sample Preparation Kit for library construction was quantified by Qubit and Q-PCR, and the Nova Seq6000 for sequencing at Novogene Co., Ltd. (Beijing, China).

### 2.9. Detection of Fecal Metabolites

The 100 mg fecal samples were ground in liquid nitrogen, placed in an EP tube, and then added 500 μL of 80% methanol aqueous solution containing 0.1% formic acid. The solution was vortexed, sonicated on ice for 30 min, centrifuged at 15,000× *g* rpm, and 4 °C for 10 min. The supernatant was diluted with mass spectrometric-grade 60% methanol-water, placed in a 0.22 μm filter tube, and centrifuged at 15,000× *g* at 4 °C for 10 min. The filtrate was collected and injected into LC-MS/MS system analysis (Vanquish UHPLC system coupled with an Orbitrap Q Exactive series mass spectrometer, ThermoFisher). The column was a Hyperil Gold column C_18_ (100 × 2.1 mm, 1.9 μm). The parameters set followed, column temperature was 40 °C, the flow rate was 0.2 mL/min, the eluents for the positive polarity mode were eluent A (0.1% formic acid in water) and eluent B (methanol), the eluents for the negative polarity mode were eluent A (5 mM ammonium acetate, pH = 9.0) and eluent B (methanol). The LC conditions were 2% B, 1.5 min; 2–100% B, 12 min; 100% B, 14 min; 100–2% B, 14.1 min; and 2% B, 16 min. Q exactive mass series spectrometer was operated in positive/negative polarity mode with spray voltage of 3.2 kV, the capillary temperature was 320 °C, sheath gas flow rate was 35 arb and aux gas flow rate was 10 arb. These metabolites were identified by the KEGG database (https://www.genome.jp/kegg/, accessed on 6 February 2021), HMDB database (https://www.hmdb.ca/, accessed on 6 February 2021) and Lipidmaps database (https://www.lipidmaps.org/, accessed on 6 February 2021). The molecular formula was predicted through molecular ion peaks and fragment ions, which were compared with mzCloud and Chemspider databases.

### 2.10. Statistical and Bioinformatics Analysis

Each of the groups’ values were expressed as the means ± standard deviation (SD), *p* < 0.05 was considered to reflect a statistically significant difference. Comparison between groups was performed using Student’s *t*-test for two groups and analyzed using one-way analysis of variance (ANOVA) for more than two groups with Tukey. The statistical significance of fecal metabolomics was calculated by univariate analysis (*t*-test), VIP > 1, *p* < 0.05 and fold change (FC) ≥ 2 or FC ≤ 0.5 of metabolites were considered to be differential metabolites.

## 3. Results and Discussion

### 3.1. The Functional Properties of SDF

As shown in Table 1, SDF had a large number of hydrophilic groups that could fully combine with water molecules and resulted in high water holding and swelling (WHC, 6.00 ± 0.3 g/g; WSC, 9.80 ± 0.2 mL/g), the WHC and WSC were higher than that of *Monascus* and *wheat bran* dietary fiber, respectively [24,25]. Therefore, consumption of SDF increased satiation to decrease food intake, lubricated the intestine, and reduced the risks of intestinal mucosal damage, enteritis and constipation. The oil-holding capacity (OHC) is an important index for evaluating the application of SDF in low-fat food, and the SDF with a good OHC (1.70 ± 0.2 g/g) can improve the characteristics of food and prolongs the shelf life of food [26]. The main absorption site of food is concentrated in the small intestine, and the intake of cholesterol and glucose positively correlates with the occurrence of coronary heart disease, arteriosclerosis and other diseases. Dietary fiber is reported to reduce blood glucose after a meal by trapping glucose and wrapping it in a network that slows the spread of glucose [27], and absorbs cholesterol by adsorbing and chelating organic molecules on the surface of cholesterol, so SDF plays a positive role in reducing intestinal cholesterol levels. The result showed that SDF had a good ability to absorb glucose and cholesterol in vitro (GAC, 24.70 ± 0.56 mmol/g; CAC, pH = 7, 5.07 ± 0.2 mg/g; CAC, pH = 2, 2.42 ± 0.5 mg/g), and the cholesterol adsorption of SDF mainly occurred in the small intestine (pH = 7.0), which was consistent with previous reports [28]. Thus, dietary SDF could absorb more cholesterol and glucose in food, and directly reduce the hyperglycemia and hyperlipidemia caused by glucose and cholesterol absorption in the blood, which is beneficial to health, such as reducing obesity and stabilizing postprandial blood glucose.

### 3.2. Monosaccharide Composition and Molecular Weight of SDF

**Figure 1:**
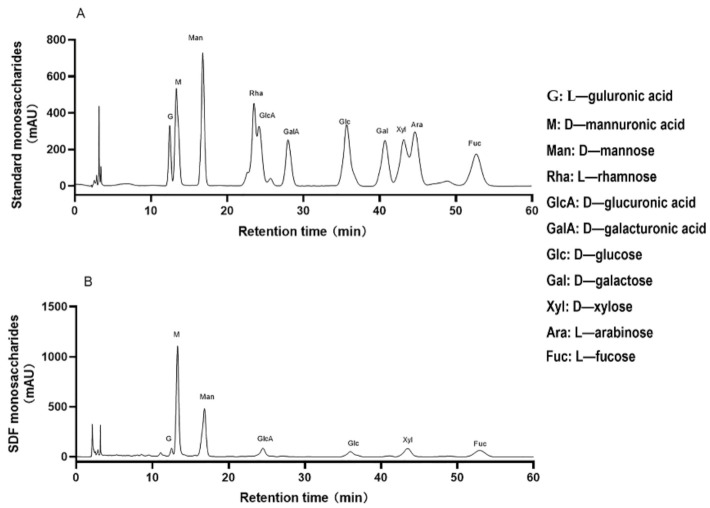
The monosaccharide composition of SDF. (**A**) was the curve of 11 standard monosaccharides, and (**B**) was the curve of SDF monosaccharides (Retention time: the time from the start of the injection of a sample component to the maximum concentration of the component behind the column).

Basic research on monosaccharide composition is essential for the further development and use of SDF. Figure 1 showed that seven monosaccharides were detected in SDF: L—guluronic acid (G, 12.50 min), D—mannuronic acid (M, 13.31 min), D—mannose (Man, 16.81 min), D—glucuronic acid (GlcA, 24.48 min), D—glucose (Glc, 35.97 min), D—xylose (Xyl, 43.48 min) and L—fucose (Fuc, 52.93 min). The results showed that the SDF was primarily composed of M and Man, and it was speculated that SDF was a soluble polysaccharide primarily composed of algin. SDF monosaccharide composition was different from the dietary fiber of terrestrial plants, such as *Pouteria glomerata* and *coconut flour* [29,30]. Algin, mostly derived from brown algae, is the main component of dietary fiber and widely used in food and medical industries. Algin absorbs the water into the gastrointestinal tract to provide moisture and facilitates defecation and stomach emptying, and it also absorbs harmful ions and glycolipids in the intestinal tract via its powerful adsorption capacity [31]. The ratio of M and G in the monosaccharide composition of SDF was 21.36, which indicated that SDF had low gel properties, good water-holding capacity and good fluidity, and it may be applied in fiber-rich beverages and oral liquids [32].

The weight-average molecular weight (Mw) was 5.277 × 104, the distribution index (PD) was 1.22, and the molecular weight distribution ratio and GPC spectrum of each stage are shown in Table 2 and Figure S1, respectively. The molecular weight of SDF was relatively low and concentrated and primarily distributed within the range of 31,519–54,000. The molecular weight of SDF obtained in this study was lower than that of other SDF according to the setting of alkaline hydrolysis conditions [33], which relatively low molecular weight was more water-soluble and more easily digested and used by intestinal bacteria.

### 3.3. SDF Inhibition of α-Amylase and α-Glucosidase Activity

**Figure 2:**
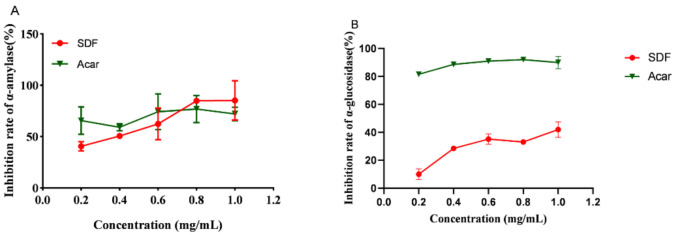
Inhibition rate of SDF and acarbose on α-amylase (**A**) and α-glucosidase (**B**). (Acar: acarbose).

Dietary fiber has an obvious glucose-lowering effect, and its mechanism may be related with the regulation of intestinal digestive enzyme activity, involving α-amylase and α-glucosidase, the key enzymes in the process of digestion [34]. Carbohydrates are first hydrolyzed into oligosaccharides by α-amylase, then decomposed into monosaccharides by α-glucosidase after entering the small intestine, which increases the postprandial blood glucose in the body. Therefore, inhibition of these enzyme activity, as an effective means, is able to generate stable blood glucose level, which is important for human health. Figure 2A showed that SDF had an increasing inhibitory rate on α-amylase activity in vitro within the range of 0.2–1 mg/mL. The inhibition rate of 1 mg/mL SDF was similar to 2.5 mg/mL pomelo fruitlet SDF [20]. There was no significant difference with acarbose, which indicated that SDF from Laminaria japonica had a strong inhibitory effect on α-amylase activity. Figure 2B showed that SDF inhibited α-glucosidase activity in vitro with increasing concentrations in the range of 0.2–1 mg/mL. Although the inhibitory activity of SDF on α-glucosidase was lower than acarbose, its inhibitory activity was approximately 15 times the activity of apricot SDF [35]. In conclusion, SDF may stabilize postprandial glucose levels by inhibiting digestive enzyme activity in a concentration-dependent manner.

### 3.4. Effects of SDF on Body Weight and Fasting Blood Glucose

**Figure 3:**
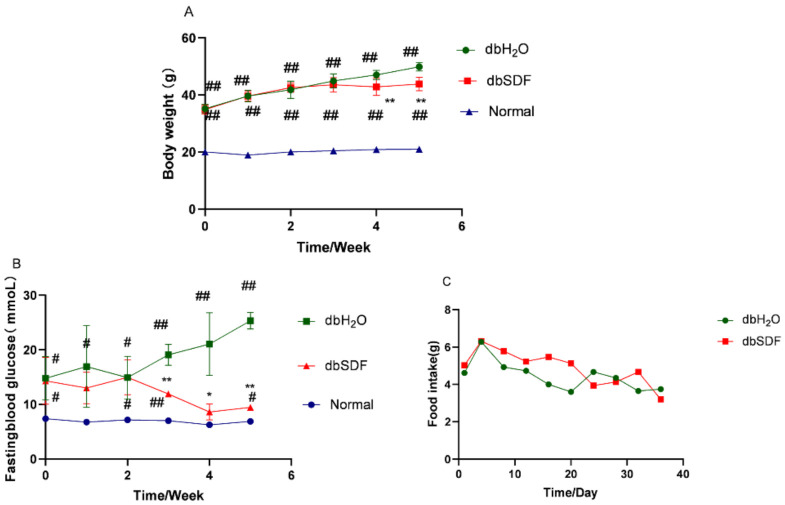
Effect of SDF on body weight (**A**), blood glucose (**B**) and food intake (**C**) of db/db mice (*n* = 6). ^#^ *p* < 0.05, ^##^ *p* < 0.01 vs. Normal group, and * *p* < 0.05, ** *p* < 0.01 vs. dbH_2_O group.

As shown in Figure 3A,B, the body weight and blood glucose of db/db mice were significantly higher than the Normal group during the entire experimental period (*p* < 0.01). However, the body weight and glucose of diabetic mice in the SDF group decreased approximately 13.32% and 62.63%, respectively, compared to the dbH_2_O group. Body weight was reduced significantly in the fourth week (*p* < 0.01) (Figure 3A) and blood glucose decreased significantly in the third week after SDF intervention (*p* < 0.01) (Figure 3B). Although SDF was reported to promote satiety and reduce food intake [36], food intake did not change significantly (5 weeks) (Figure 3C), which may be due to the inability of db/db mice to feel satiated. These results showed that SDF reduced the weight gain and blood glucose of diabetic mice, but not via the regulation of food intake, which is consistent with the results of *Fuzhuan brick* tea polysaccharides [37]. Combined with the results of knot 3.1–3.3, it is speculated that SDF may reduce blood glucose and body weight by promoting gastrointestinal peristalsis, accelerate gastric emptying and the absorption of the sugars and lipids in food.

### 3.5. Effects of SDF on Insulin Resistance and Lipids

**Figure 4:**
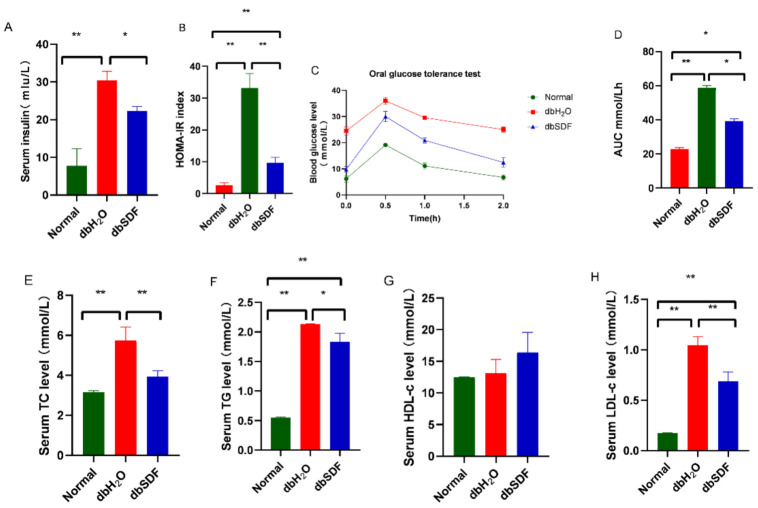
Effect of SDF on insulin resistance and serum lipids in db/db mice. (**A**) Insulin (INS). (**B**) HOMA-IR index. (**C**) glucose tolerance curve. (**D**) the area under the curve. (**E**) Total cholesterol (TC). (**F**) Triglycerides cholesterol (TG). (**G**) High-density lipoprotein cholesterol (HDL-c). (**H**) Low-density lipoprotein cholesterol (LDL-c), (*n* = 6). * *p* < 0.05, ** *p* < 0.01.

Db/db mice have an uncontrolled appetite from birth and developed severe insulin resistance associated with hyperinsulinemia, hyperglycemia, and hypertriglyceridemia [38], and these phenomena are consistent with the results in Figure 4A–H. The serum insulin level and homeostasis model assessment for insulin resistance (HOMA-IR) index are important indicators of insulin resistance in the body. The serum insulin content of the dbH_2_O group was nearly four times that of the Normal group in Figure 4A, and the HOMA-IR index increased significantly (*p* < 0.01) (Figure 4B), which indicated severe insulin resistance in the dbH_2_O group. However, SDF significantly reduced the insulin content and alleviated insulin resistance in db/db mice (*p* < 0.01). Glucose tolerance and insulin resistance are closely associated with obesity, which may be measured by the oral glucose tolerance test (OGTT). As shown in Figure 4C, the OGTT results demonstrated that the level of blood glucose reached a maximum at 0.5 h and recovered 2 h after a glucose challenge. A single administration of SDF reduced the glycemic response compared to the dbH_2_O group. The area under the curve (AUC) above baseline glucose was shown in Figure 4D. Compared to the Normal group, the AUC of the OGTT value for dbH_2_O was significantly increased to a high degree of certainty (*p* < 0.01). However, the AUC of the OGTT value for SDF treatment was significantly lower (*p* < 0.01) than the dbH_2_O group, which was similar to that *Bamboo shoot* shell derived dietary fiber improves glucose tolerance in diabetic mice, suggesting that SDF attenuated insulin resistance in db/db mice [39]. The levels of TG, TC, and LDL-c in the dbH_2_O group were extremely significantly higher than the Normal group (*p* < 0.01), and TC (*p* < 0.01), LDL-c (*p* < 0.01) and TG (*p* < 0.05) were significantly reduced in the SDF intervention group compared to the dbH_2_O group (Figure 4E,F,H), which demonstrated that SDF regulated the disordered blood lipid metabolism. HDL-c is a protective factor against atherosclerosis and coronary heart disease [40]. However, the HDL-c content in the dbSDF group compared with a 24.94% increase for the dbH_2_O group, which suggested that the effects of SDF were attributed to lipid regulation (Figure 4G).

### 3.6. Effects of SDF on Intestinal Flora

The coverage values of the dbH_2_O, dbSDF and Normal groups were 99.96%, 99.97% and 99.96%, respectively, which were used to evaluate the integrity of sampling. The results elucidated that in the present samples, more than 99% of bacterial system were identified. In total, 615,540 raw tags were obtained by Illumina NovaSeq sequencing, 630,268 clean tags were filtered by splicing and quality control, and 579,889 effective tags were used for subsequent analysis after chimera filtering. A total of 381, 312 and 319 OTUs were detected in the dbSDF, Normal and dbH_2_O groups, respectively. Alpha diversity is applied in analyzing the species richness and uniformity, in which Chao1 reflects species richness and Shannon reflects species diversity between groups [41]. Although most studies suggested that higher abundance and diversity of intestinal flora were better [42], our experimental results showed that the Chao1 (298.87 ± 24.13) and Shannon (2.47 ± 0.45) of the dbH_2_O group were greater than the Chao1 (269.14 ± 49.20) and Shannon (2.01 ± 0.53) of the Normal group (Table S1). The richness and diversity of db/db obese mice flora were higher than Normal group, which may be caused by the increase in the flora related to metabolic diseases, such as diabetes and obesity. After 5 weeks of SDF intervention, Chao1 (285.86 ± 30.99) and Shannon (2.18 ± 0.43) in the dbSDF group were reduced compared to the dbH_2_O group, and suggested that SDF changes the composition and richness of the host flora, which were similar to the regulation trend of intestinal flora by SDF from soybean residue [43]. Principal coordinate analysis (PCoA) of the intestinal flora of db/db mice based on unweighted and weighted unifrac distance methods was depicted in Figure S2, which also showed that the clustering positions of the dbSDF group and the Normal group were closer than the dbH_2_O group and the Normal group. Therefore, the composition and structure of the intestinal flora was more similar to Normal group after 5 weeks of SDF intervention compared to the dbH_2_O group, which was consistent with the changing trends of blood sugar, blood lipids and body weight. To analyze the relationship between the composition of the intestinal flora and the improvement of diabetes after SDF treatment, Spearman correlation analysis between the intestinal flora and the indicators of glucose and lipid metabolism disorders was shown in Figure S3A. This result further indicates that the hypoglycemic mechanism of SDF is related to improving intestinal flora and regulating glucose and lipid metabolism.

**Figure 5:**
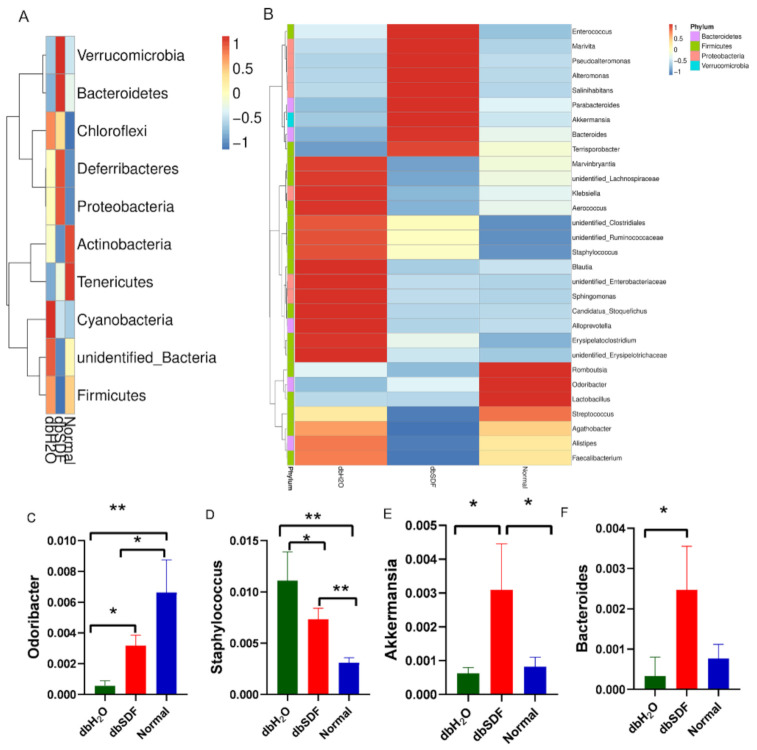
Composition of gut flora in three groups. (**A**), heat map of relative abundance of top10 phyla level. (**B**), clustering map of the top 30 species abundance at the genus level. (**C**–**F**), the columns abundance of *Odoribacter*, *Staphylococcus*, *Akkermansia* and *Bacteroides, respectively* (*n* = 3). * *p* < 0.05, ** *p* < 0.01.

Firmicutes and Bacteroidetes were related to obesity and diabetes [44]. The abundance of Firmicutes in the dbSDF group was significantly lower than the dbH_2_O group, and the abundance of Bacteroidetes increased significantly, which were consistent with SDF results derived from *Beet Pulp* [45], but the ratio of Firmicutes and Bacteroidetes was not significantly changed (Figure 5A), which may be caused by the short experimental time. After SDF intervention, the species abundance of intestinal flora of mice in each group changed significantly, as shown in Figure 5B. SDF increased the abundance of butyric acid-producing bacteria, such as *Odoribacter* (*p* < 0.05) [46] and *Akkermansia* (*p* < 0.01) (Figure 5C,E) [47]. Butyric acid is produced by the fermentation of cellulose in the gut flora of humans and rodents, which maintains and repairs intestinal mucosal health and barrier, and reduces insulin resistance and inflammation [48,49]. *Akkermansia* has been extensively studied in recent years, and its abundance is inversely associated with body weight and diabetes risk, and this genus is considered a symbol of intestinal health [50]. Other beneficial bacteria increased by SDF were *Bacteroides* (*p* < 0.05) (Figure 5F), which contain genes responsible for dietary fiber substitution that are not present in the human genome, so it could contribute to the degradation of polysaccharides in the intestinal tract [51,52]; moreover, the number of *Bacteroides* in the intestinal flora of obese mice is decreased compared to lean mice [53]. The db/db mice are spontaneously obese mice hyperglycemia, so the abundance of *Odoribacter*, *Bacteroides* and *Akkermansia* in the dbH_2_O group significantly lower than the Normal group, but the abundance in the dbSDF group was significantly higher than the dbH_2_O group after 5 weeks of treatment. In combination with the changes of body weight and blood glucose level of mice, and it followed that the SDF may achieve the goal of weight loss by increasing the abundance of beneficial bacteria. In addition to increasing the beneficial bacteria, SDF also reduced the abundance of harmful *Staphylococcus* (*p* < 0.05) (Figure 5D), which is a common Gram-positive bacterium that induces infection. The increased abundance of *Staphylococcus* induces intestinal inflammation, diarrhea and other intestinal diseases, and it also infects the extracellular domain of the insulin-binding protein by secreting LTAS, which prevents insulin-mediated glucose uptake and leads to impaired glucose tolerance [54]. This finding supported the finding that SDF improved the intestinal immunity of mice, increased glucose intake and alleviated impaired glucose tolerance by reducing the abundance of *Staphylococcus*.

### 3.7. Effects of SDF on Intestinal Metabolites

**Figure 6:**
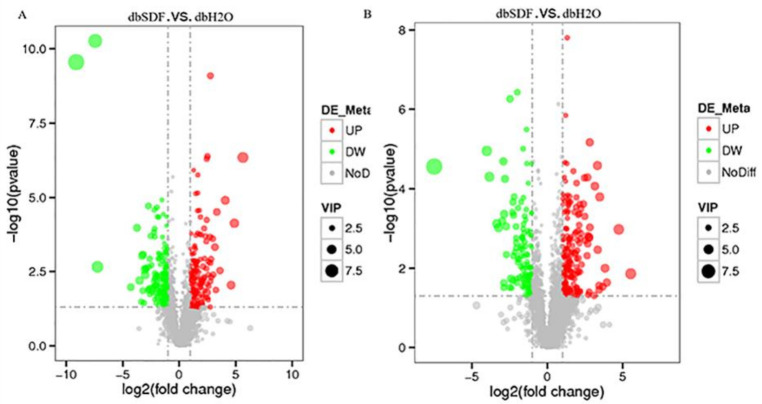
Differential metabolite volcano map. (**A**), positive ion mode, (**B**), negative ion mode. The abscissa represented the expression fold change of metabolites in different groups (log_2_ fold change), the ordinate represented the significance level of difference (−log_10_ *p*-value), each point in the volcano graph represented a metabolite, and the significantly up-regulated metabolites were marked with red points, the significantly down-regulated metabolites were represented by green dots, and the size of dot represented the VIP value.

The Pearson correlation coefficients between QC samples were calculated as R^2^ > 0.98. Principal component analysis (PCA) showed that the differences of quality control samples were small, the overall metabolic difference among samples of each group was large, and the intra-group variability was lower (Figure S4). The above descriptions demonstrated that the entire detection process was stable, and the data were of good quality. SDF is not digested by the body, but is used by flora in enterology, producing a small amounts of energy and various bioactive substances [55]. The 3101 and 2177 metabolites were detected using the positive and negative ion model methods, respectively, and the total differential metabolites were 305 and 319, respectively. A total of 188 and 158 were significantly increased, and 177 and 161 were significantly decreased, respectively. Changes in intestinal metabolites are closely related to the health of the body [56], and the overall distribution of differential metabolites intuitively reflected that SDF greatly changed intestinal metabolites, as shown in Figure 6, which was consistent with changes in the intestinal flora.

**Figure 7:**
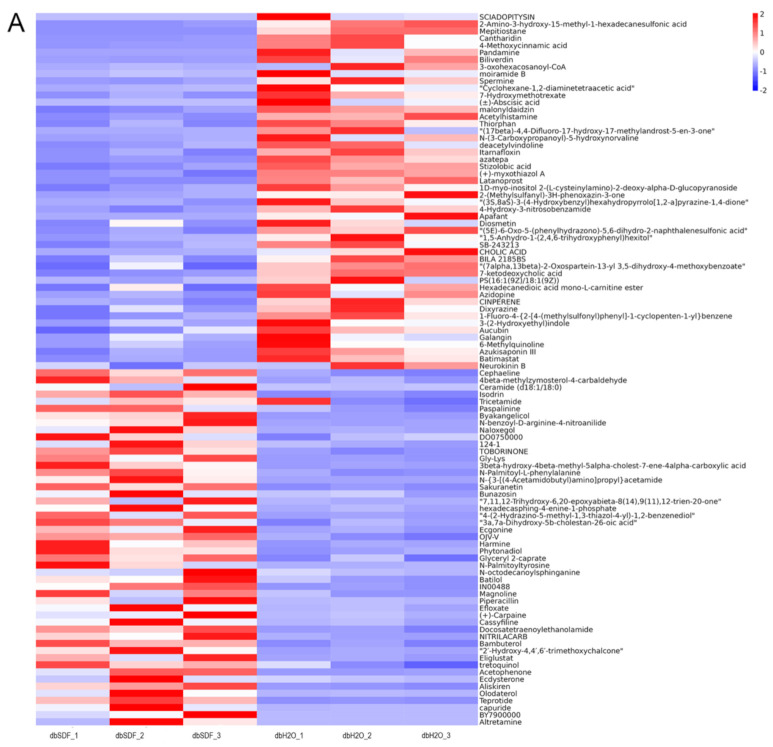

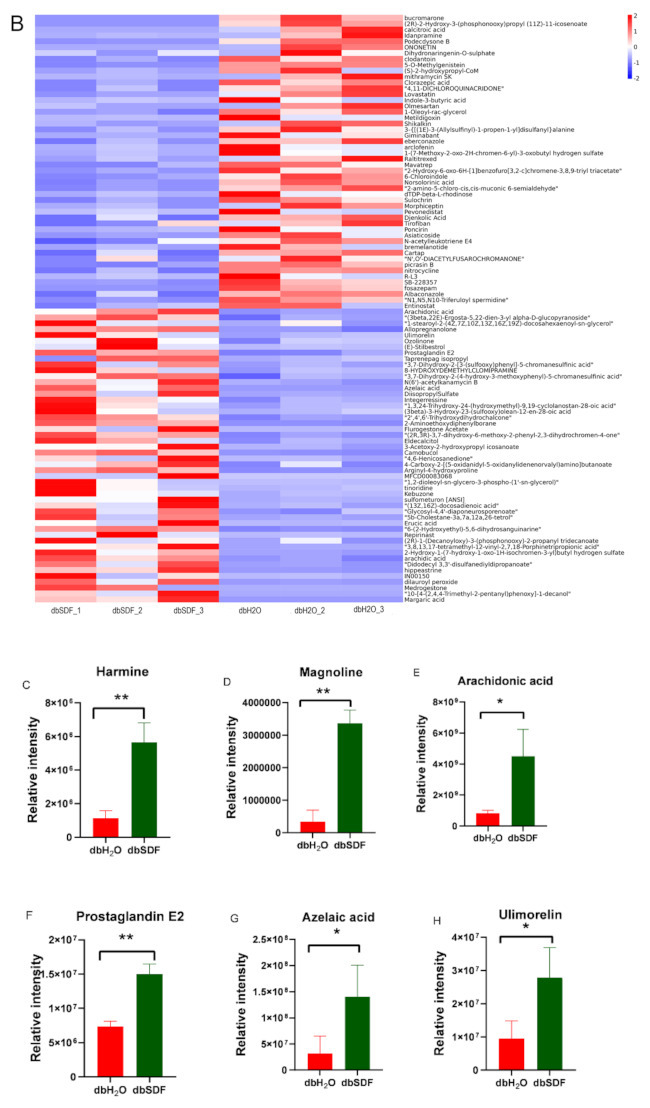
Different compositions of metabolites. (**A**) differential metabolite clustering heat map in positive ion mode, (**B**) differential metabolite clustering heat map in negative ion mode. The vertical direction was the clustering of samples, and the horizontal direction was the clustering of metabolites. (**C**) the abundance of hamin. (**D**) the abundance of magnoline. (**E**) the abundance of arachidonic acid. (**F**) the abundance of prostaglandin E2. (**G**) the abundance of azelaic acid. (**H**) the abundance of ulimorelin. (*n* = 3). * *p* < 0.05, ** *p* < 0.01.

The metabolites with significant changes in feces were depicted in Figure 7A,B. There were significant differences in the cluster of metabolite abundance in mice of dbH_2_O, dbSDF and Normal groups, which indicated that the composition of intestinal metabolites was significantly changed after SDF intervention. Most studies on the relationship between dietary fiber and the intestine focused on short-chain fatty acids, but other metabolites may also play a vital role. In order to observe the degree of change directly, we made an important analysis on substances that are beneficial to lower blood sugar and relieve intestinal inflammation. The abundances of harmine, magnoline, arachidonic acid, prostaglandin E2, azelaic acid and ulimorelin were significantly increased with SDF treatment, as shown in Figure 7C–H, and the correlation with genus-level flora was determined using Spearman correlation analysis, as shown in Figure S3B. Harmine is a cell type-specific regulator that regulates the expression of peroxisome proliferator-activated receptor gamma (PPARγ) by inhibiting of the Wnt signaling pathway. PPARγ is a central regulator of adipocytes, and it increases the expression of fatty acid oxidation genes, energy metabolism and heat dissipation genes, thus increasing the energy consumption of the body and reducing fat accumulation [57,58]. Harmine can also induce β cell proliferation to improve islet quality and blood glucose control, and its anti-inflammatory, anti-bacterial and anti-viral activities have been extensively studied [59,60]. Therefore, SDF increased the content of harmine in the intestinal tract, which was closely related to the function of SDF in anti-inflammatory, lipid-lowering and blood glucose reduction. The arachidonic acid metabolite prostaglandin E2 enhances the production of anti-inflammatory cytokines and inhibits the production of pro-inflammatory cytokines [61], but the role of arachidonic acid metabolites in inflammation is not an isolated process. It is closely related to other inflammatory mediators and participates in the development of pathophysiology. Therefore, studies should pay attention to the organic connection of various media to reveal the law and mechanism of biological activities more deeply. Ulimorelin is a small molecule growth hormone agonist and prokinetic agent that increases the frequency of bowel movements. Ulimorelin and its related compounds may effectively fight constipation through good control of the intestinal nerves [62], help to reduce the stay time of food in the intestine and the absorption of nutrients, which was of great significance for the control of postprandial blood glucose. Azelaic acid is found naturally in plants, animals and humans, and it has an anti-inflammatory effect by inhibiting ROS production by neutrophils [63]. Magnoline had anti-inflammatory and protective effects on the kidneys of diabetic rats [64]. Therefore, these metabolites produced by the fermentation of SDF by intestinal flora, as chemical messengers of the interaction between flora and host, play an important role in the regulation of diabetes treatment, such as anti-inflammatory, improved gastrointestinal motility and lowering blood glucose. The bubble diagram of the KEGG pathway of intestinal metabolites of TOP 20 was shown in Figure S5, and the major metabolic pathways involved were bile secretion, biosynthesis of unsaturated fatty acids, primary bile acid biosynthesis, inflammatory mediator regulation of TRP channels, regulation of lipolysis in adipocytes and cholesterol metabolism in negative ion model, in additional, metabolic pathways, pyrimidine metabolism, arginine and proline metabolism, carbon metabolism, glycine, serine and threonine metabolism, glycolysis/gluconeogenesis, citrate cycle (TCA cycle), pyruvate metabolism, starch and sucrose metabolism in positive ion model. KEGG pathway analysis is closely related to biological phenomena, reflecting the interactions between molecules, which lay a foundation for subsequent data mining.

## 4. Conclusions

SDF derived from *Laminaria japonica* had good water absorption, oil retention, glucose and cholesterol adsorption, and intestinal digestive enzyme inhibition capacity, which laid a good foundation for subsequent mouse experiments and applications. Therefore, SDF effectively reduced body weight, blood glucose, and blood glucose and insulin resistance in diabetic mice after 5 weeks of intervention. The mechanism of SDF in alleviating diabetes mainly focuses on intestinal flora and metabolites. The results demonstrated that SDF significantly changed the intestinal flora structure and metabolite composition, increased the abundance of the beneficial bacteria *Akkermansia*, *Odoribacter* and *Bacteroides* and reduced the abundance of the harmful bacterium *Staphylococcus*. The relative contents of beneficial products, such as harmine, magnoline, arachidonic acid, prostaglandin E2, ulimorelin, and azelaic acid, were increased. Consequently, SDF shows that intervention of the intestinal flora and metabolites has broad prospects in the prevention and treatment of T2DM. However, the little essential understanding for probiotics good bacteria in the intestine and the complexity of functional metabolites leads to the little development of the theory. However, it will, no doubt, become an important part of future research. This research lays a foundation for improving the utilization rate of *Laminaria japonica* resources and the economic value of *Laminaria japonica* culture and promotes functional research and the application of dietary fiber.

## Figures and Tables

**Table 1:** The functional properties of SDF (Data were expressed by means ± standard deviation).

WHC (g/g)	OHC (g/g)	WSC (mL/g)	GAC (mmol/g)	CAC (mg/g) pH = 7	CAC (mg/g) pH = 2
6.00 ± 0.3	1.70 ± 0.2	9.80 ± 0.2	24.70 ± 0.56	5.07 ± 0.2	2.42 ± 0.5

WHC: Water_holding capacity; OHC: Oil_holding capacity; WSC: Water_swelling capacity; GAC: Glucose absorption capacity; CAC: Cholesterol adsorption capacity.

**Table 2:** The molecular weight distribution of SDF.

Molecular Weight Distribution (MWD)	Proportion
31,519–54,000	73.50%
54,000–115,000	22.70%
115,000–185,000	3.00%
185,000–41,497	0.80%
31,519–54,000	73.50%

## Data Availability

Not applicable.

## Supplementary Materials

The following supporting information can be downloaded at: https://www.mdpi.com/article/10.3390/nu14020329/s1, Figure S1: The GPC spectrum of SDF (the red line was LS detector, blue line was dRI detector); Figure S2: Two dimensional Principal Co-ordinates Analysis (PCoA); Figure S3: Spearman correlation heatmap of top 35 gut microbes genus and HOMA-IR, TG, TC, HDL-c, LDL-c and INS; Figure S4: PCA analysis of the total sample; Figure S5: The KEGG enrichment bubble chart; Table S1: Alpha diversity analysis of Normal, dbSDF and dbH_2_O group.

## Abbreviations

SDF, soluble *Laminaria japonica* dietary fiber; T2DM, diabetes mellitus type 2; TG, Triglycerides cholesterol; TC, total cholesterol; HDL-c, high-density lipoprotein cholesterol, LDL-c, low-density lipoprotein cholesterol; INS; insulin; dbH_2_O, model control group; Normal, normal control group; dbSDF, soluble *Laminaria japonica* dietary fiber group; HOMA-IR, homestasis model assessment for insulin resistance. G, L—guluronic acid; M, D-mannuronic acid; Man, D—mannose; Rha, L—rhamnose; GlcA, D—glucuronic acid; GalA, D—galacturonic acid; Glc, D—glucose; Gal, D—galactose; Xyl, D—xylose; Ara, L-arabinose; Fuc, L—fucose; WHC, Water-holding capacity; OHC, Oil-holding capacity; WSC, Water-swelling capacity; GAC, Glucose absorption capacity; CAC, Cholesterol adsorption capacity; Acar, acarbose.
