# Supplementary material for: Physicochemical Properties of the Soluble Dietary Fiber from Laminaria japonica and Its Role in the Regulation of Type 2 Diabetes Mice
Source: Nutrients. 2022 Jan 13;14(2):329. doi: 10.3390/nu14020329 (PMC8779286; doi:10.3390/nu14020329)
Supplement: Supplementary file 1 [file nutrients-14-00329-s001.zip › Supplementary/Supplement table.pdf]

nutrients MDPI

Supplementary Table S1. Alpha diversity analysis of Normal, dbSDF and dbH_2O group.

	Group		
Alpha diversity analysis	Normal	dbSDF	dbH_2O
Chao1	269.14±49.20	285.86±30.99	298.87±24.13
Shannon	2.01±0.53	2.18±0.43	2.47±0.45
