# Supplementary figures and images for: Physicochemical Properties of the Soluble Dietary Fiber from Laminaria japonica and Its Role in the Regulation of Type 2 Diabetes Mice
Source: Nutrients. 2022 Jan 13;14(2):329. doi: 10.3390/nu14020329 (PMC8779286; doi:10.3390/nu14020329)

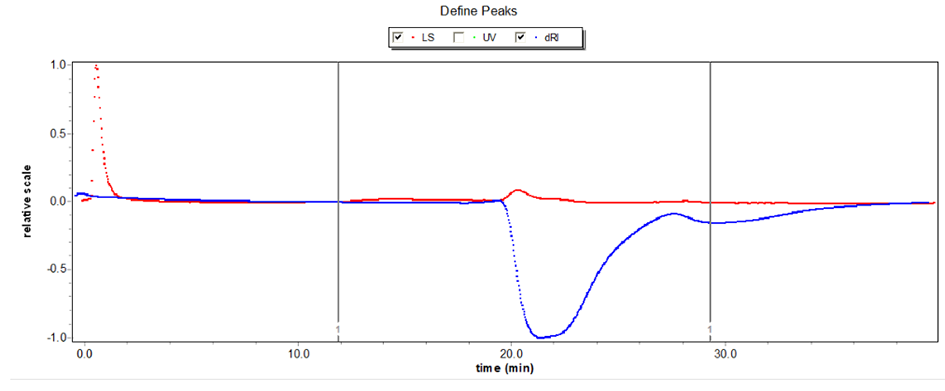

Supplement: Supplementary file 1 [file nutrients-14-00329-s001.zip › Supplementary/Supplementary figure S1.tif]

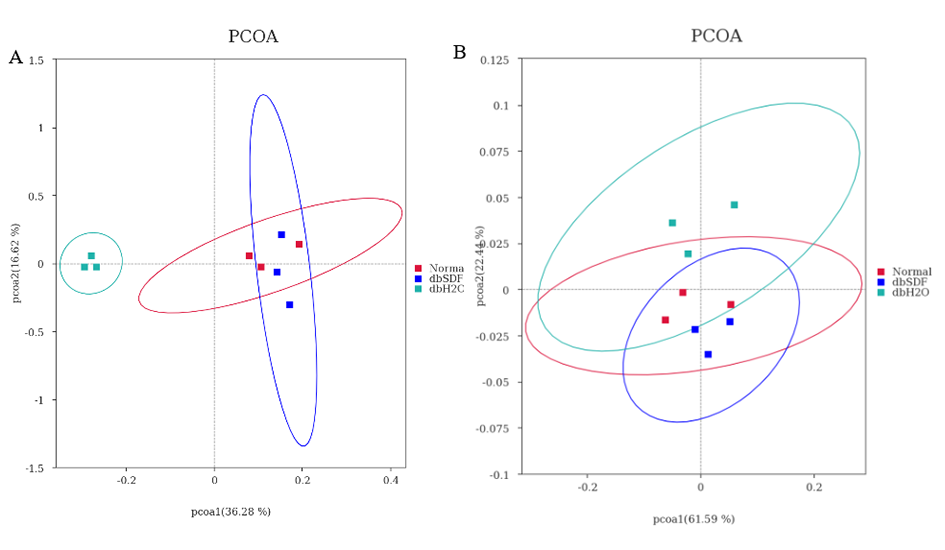

Supplement: Supplementary file 1 [file nutrients-14-00329-s001.zip › Supplementary/Supplementary figure S2.tif]

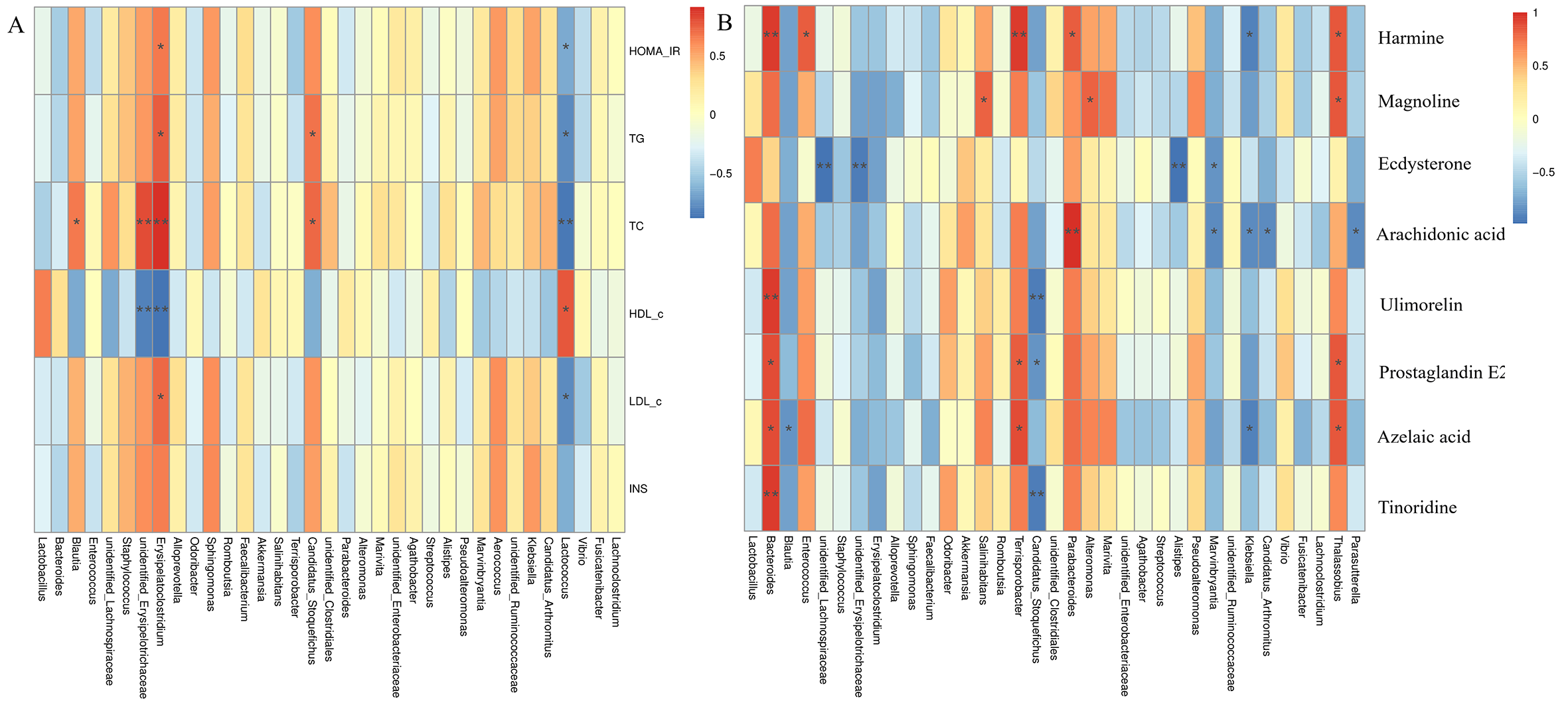

Supplement: Supplementary file 1 [file nutrients-14-00329-s001.zip › Supplementary/Supplementary figure S3.tif]

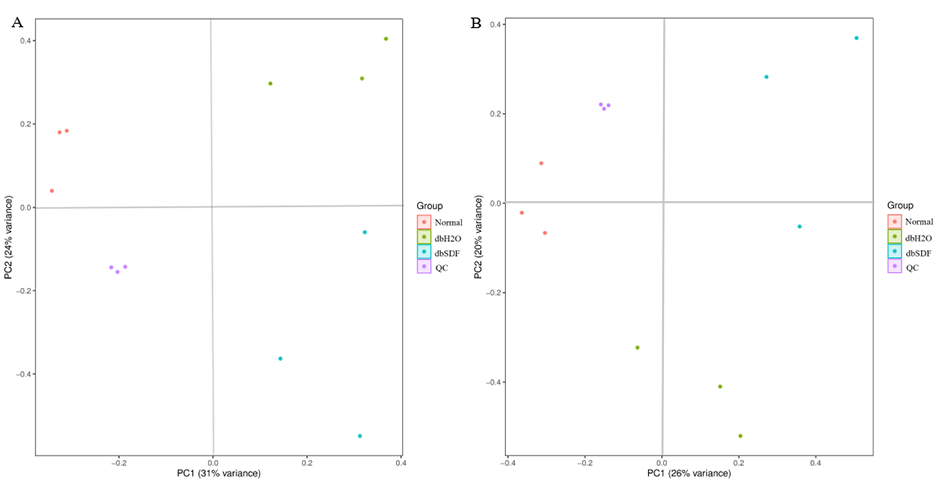

Supplement: Supplementary file 1 [file nutrients-14-00329-s001.zip › Supplementary/Supplementary figure S4.tif]

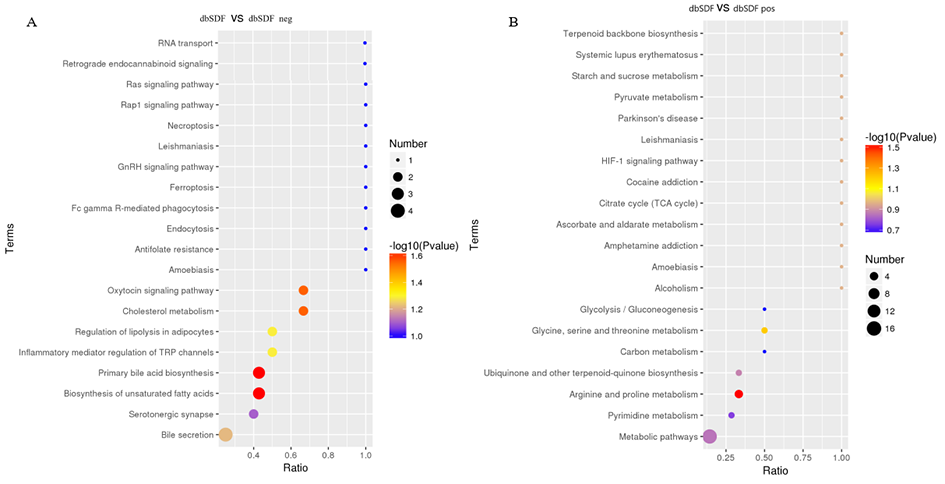

Supplement: Supplementary file 1 [file nutrients-14-00329-s001.zip › Supplementary/Supplementary figure S5.tif]
